# Professional Mental Instability and Its Dangers

**Published:** 1913-08-16

**Authors:** 


### THE NATIONAL MEDICAL GUILD.

President: CHAS. BUTTAR, M.A., M.D., B.C.

Gen. Sec.: P. C. RAIMENT, M.R.C.S., L.R.C.P.

Offices: 34 Villiers Street, Strand, London, W.C.

### PROFESSIONAL MENTAL INSTABILITY AND ITS DANGERS.

The instability of the mind of the profession at the present moment was clearly demonstrated at Brighton by the vote of the representatives on the subject of trade unions. Fifty-three voted for an amendment favourable to unionism, and sixty-seven cast their votes against, very nearly a division of half and half. And this result is typical of the mind of the profession. At the moment a beaten and discredited body, it is difficult to get two men to agree on any single point, or to follow any line of policy. If any good is to accrue to the profession in the future this indecisiveness will have to cease. The members must be united on one platform and one policy, and be prepared to give unswerving allegiance to the men whom they have chosen to lead them.

These conclusions must be patent to every trained observer of the medico-political horizon, and will not be disputed by those practitioners who are also men of affairs. When, however, the suggestion of wise solution is demanded the difficulties to be faced become apparent. So far the problem has not been solved, although some of the clearest thinkers in the profession have turned their minds to it, and the suggestions made are various if not convincing.

### Financially Unsound and Impossible.

First and foremost is the plan of the British Medical Association, which has submitted several schemes, the chief one being that prepared by the State Sickness Insurance Committee, and associated particularly with the name of Dr. Beaton. This is a scheme for raising money to a large amount which shall be at the disposal of the profession in any future dispute. In order to create this capital fund, it is proposed to levy a voluntary subscription of £5 per member per annum, which shall be paid into a central fund. We have before us as we write the imaginary balance sheet of the proposed fund for the first year, in which the Committee budget on a basis of 10,000 subscribers, a rather liberal estimate, be it remarked. This will produce a sum of £50,000 per annum. So far so good. Now what is to be done with the money so collected? The first charge is a grant to each local medical committee, some 240 in number, of £100 per annum for their working expenses. This accounts for nearly half the fund, or £24,000. Legal expenses take another £1,000, so that half the capital fund disappears at once, leaving only 50 per cent., or £25,000. Is this to be carried to reserve? Not at all. Four organising secretaries and their travelling expenses take another £3,400; maintenance of representatives at representative meetings and maintenance of members attending committee meetings in London a trifle of £4,000. The committee set up to administer the fund collect £500, the State Sickness Insurance Committee (possibly as an award for evolving so brilliant a scheme) takes £1,000, and, finally, the clerical staff of the Association situated in London, Edinburgh, Dublin, and Cardiff absorb a further £3,000, making together a total of £11,900. This leaves an estimated balance to be placed to reserve of only £13,000 per annum, or some 25 per cent. of the sum raised. It follows that aften ten years, out of half a million of money subscribed by the profession, they may have a sum of £130,000 standing to their credit to be used in any emergency that may arise!

We ask the State Sickness Insurance Committee, in all seriousness, Do they really think that the members of the medical profession will accept such ruinous finance? Does the profession require a scheme that costs 13s. 4d. to collect £1? We venture to think that the answer is in the negative, and that the scheme is dead at its birth. This, then, is the panacea of the British Medical Association, and presumably this is their method of dealing with the future of the profession. If this be indeed so, the reputation of the British Medical Association as a statesmanlike and businesslike organisation must crumble to pieces.

### The Proposals of the Medical Guild.

The National Medical Guild has also put forward a scheme for dealing with the future, and it is this: As a trade union they have power to levy funds from their members for any particular or general purpose; such funds must be free of administration charges, saving only the actual cost of collecting the money. The amount and the uses to which such levy shall be put are determined by the members through their Council, and if a levy is duly made in the form prescribed by law the member so levied *must* pay unless the Council give him exemption. Herein lies the difference between the Association and the Guild in dealing with this matter. Voluntary effort as proposed collects money only from the willing member, who has already dipped his hand pretty freely in his pocket in the past two years; collection of funds through the Guild makes everyone contribute unless he can show good cause why he should not. But it will be objected that the unwilling, knowing this, will not join and will so go scot free. That is quite true, but our endeavour is to get in so large a proportion of the willing members to join us that the other variety will see that it is to their interest to come inside our organisation. This is a large proposition and will take time to achieve, and it will be our constant task, in season and out, to impress on the profession the means by which this end may be attained.

### Unity at Leicester: Its Strength and Authority.

If one will carefully read the speech of Dr. Wallace Henry, the chairman of the Leicester Union of Practitioners, as reported in the Supplement of the *Journal* of July 26, he will find that Dr. Henry is fully alive to the importance of making it to the direct interest of the profession to become and to remain members of their trade union, and he proposes to do this by making membership of the union a necessary preliminary to admission to the staff of the Public Medical Service. It is a matter of common knowledge that the Medical Service of Leicester is the most highly organised of any in the country, and this is largely the result of the enterprise and public spirit of the local profession, to all of whom we owe a debt of gratitude for their example. This right to be a member of the Public Medical Service is a very valuable one in Leicester, and, as Dr. Wallace Henry pointed out, would act as a powerful deterrent to anyone acting contrary to the declared policy of the union. Leicester is only a small unit of the profession, but what they have done it is possible for the profession in other towns to do. What is required is a man of energy and business capacity to organise the profession in the first instance and the rest follows.

This then is one method of giving the profession a direct financial interest in their professional organisation, and the National Medical Guild in their rules have taken powers to set up and work a Public Medical Service for the non-insured population in any area that desires it. As we stated last week a tentative attempt is being made in one London Borough and the scheme will be adapted for any locality that desires to organise their local contract work on similar lines. Should any such read these lines it is to be hoped that they will communicate with us and we will give them all the assistance in our power.

### Sickness and Accident Funds and Insurance.

The other method of giving the profession a material interest in their organisation is by the establishment of (*a*) sickness and accident funds, and (*b*) life insurance. This we at once freely admit can be conducted on co-operative lines by any organised body, and the British Medical Association has already put forward some tentative proposals on the subject. But there is this one difference: the Guild has powers to allocate some of its reserve funds to this work, and it could do so in two ways. First, either by paying the whole or a portion of the premium or by increasing the amount of the policy without further cost to the insured, and the Guild means to devote such sums as it can afford to so doing.

It therefore remains for the profession to decide on what lines it wishes to proceed in the future. If it desires on the one hand to raise funds for its protection which shall be free from liability to attachment, and free from enormous administration expenses it will come into the Guild or some other trade union; if, on the other hand, it prefers to run all the legal risks that it has run in the past, it will remain where it is. It is for the profession to decide.

### What Does the Medical Profession Desire?

At the commencement of this article we remarked on the instability of the mind of the profession at this juncture, and it may not be out of place again to ask the question, "What does the profession desire?" The organisers of the Guild believe, so far as the present situation is concerned, that there is no hope of anything being done in the immediate future to alleviate it, but that this period should be regarded as a time of preparation for the next struggle that will come upon us sooner than later. If the next battle is to be fought successfully we must find some common platform on which we can unite our differences and then proceed with our organisation to effect our object. At the present moment the profession is smarting under defeat and ill-usage. This period of apathy must and will cease, and clarity of thought succeed the present confusion of ideas. Until that confusion has definitely crystallised into a common policy no active steps can be taken to put the profession in a better position, or to prevent it falling into a worse one. At the present moment, therefore, we are content to preach the broad outlines of trade unionism as applied to medicine rather than attempt to put forward a concrete policy; nevertheless, that policy is being slowly matured as the profession shows more clearly what it desires.

### A Definite Advantage for Members.

The lines on which we propose to move in the autumn are those that will carry out some of the ideas that we have been putting forward from week to week, and our first attempt will be in the direction of giving our members a definite advantage out of their membership on the lines we have mentioned. The first work that we find ready to our hands is the organisation of all forms of contract practice on the non-insured person, bearing in mind that the uninsured of to-day will be the insured of to-morrow. From that we shall proceed to develop our plan of sickness and pension insurance and so give the member a direct financial interest in the Guild.

### The Organisation of Contract Practice.

One word about the organisation of contract practice. It is a large subject to tackle, but we feel confident that there is an answer to the problem, and the key to the whole situation is the unity of the profession. Is unity hopeless? We venture to think not, and can point to Leicester as a forcible example. What Leicester can do, Plymouth or Southampton can achieve; and, although there are difficulties ahead in tackling London, yet the same key-unity will unlock the casket that contains the secret of success. Hence, we do not despair of finding that key in our search, provided that we have the help of a willing band of workers, content to drop self in the achievement of the aim they have in hand.

